# Dimethyl sulfoxide affects the viability and mineralization activity of apical papilla cells in vitro

**DOI:** 10.1590/0103-644020246054

**Published:** 2024-12-16

**Authors:** Letícia Martins Santos, Danielle Yumi Shimabuko, Carla Renata Sipert

**Affiliations:** 1 Department of Biomaterial and Oral Biology, School of Dentistry, University of São Paulo, São Paulo, Brazil; 2 Department of Restorative Dentistry, School of Dentistry, University of São Paulo, São Paulo, Brazil.

**Keywords:** Dimethyl sulfoxide, Cytotoxicity, Cell mineralization activity, Apical Papilla Cells

## Abstract

Dimethyl sulfoxide (DMSO) is widely used as an adjuvant in dissolving insoluble compounds in an aqueous medium; however, it can induce significant molecular changes in cells. The possible damages may occur obeying a tissue-specific profile, and the effect on human apical papilla cells (hAPC) remains unknown. Therefore, this study aimed to evaluate DMSO effects on the viability and mineralization activity in hAPC cultures in vitro and to establish standards of maximum concentrations for its use in laboratory routines. hAPCs were cultured, plated, and maintained in media containing increasing concentrations of Dimethyl sulfoxide (0.1%, 0.5%, 1%, 5%, and 10%) for 24 h, 48 h, 72 h, and 7 days. At each time point, the cells were subjected to the MTT assay. The Alizarin red S staining assay was performed to evaluate the osteo/odontogenic mineralization potential of hAPC DMSO-exposed (0.1%, 0.5%, and 1%) in the 21-day time-point. Statistical analysis was performed using one-way analysis of variance followed by Tukey's post hoc test (p<0.05). In general, the 5% and 10% DMSO concentrations were shown to be cytotoxic for hAPC at all analyzed time points, and the hAPC DMSO-stimulated presented higher osteo/odontogenic mineralization potential. Therefore, the 5% and 10% DMSO concentrations should be avoided, and the mineralization activity assay should be carefully designed in order to avoid biases at in vitro assays using hAPC cultures.

## Resumo

O dimetilsulfóxido (DMSO) é amplamente utilizado como adjuvante na dissolução de compostos insolúveis em meio aquoso; no entanto, pode induzir alterações moleculares significativas nas células. Os possíveis danos podem ocorrer obedecendo a um perfil específico do tecido, e o efeito nas células da papila apical humana (hAPC) permanece desconhecido. Portanto, este estudo teve como objetivo avaliar os efeitos do DMSO na viabilidade e atividade de mineralização em culturas de hAPC in vitro e estabelecer padrões de concentrações máximas para seu uso em rotinas laboratoriais. As hAPCs foram cultivadas, plaqueadas e mantidas em meio contendo concentrações crescentes de dimetilsulfóxido (0,1%, 0,5%, 1%, 5% e 10%) por 24 horas, 48 horas, 72 horas e 7 dias. Em cada momento, as células foram submetidas ao ensaio MTT. O ensaio de coloração Alizarin red S foi realizado para avaliar o potencial de mineralização osteo/odontogênica das hAPC expostas ao DMSO (0,1%, 0,5% e 1%) no período de 21 dias. A análise estatística foi realizada por meio de análise de variância unidirecional seguida pelo teste post hoc de Tukey (p<0,05). Em geral, as concentrações de 5% e 10% de DMSO mostraram-se citotóxicas para hAPC em todos os momentos analisados e as hAPC estimuladas por DMSO apresentaram maior potencial de mineralização osteo/odontogênica. Portanto, as concentrações de 5% e 10% de DMSO devem ser evitadas, e os ensaios de atividade de mineralização celular devem ser cuidadosamente elaborados para evitar vieses nos ensaios *in vitro* utilizando culturas de hAPC.

## Introduction

Stem Cells from Apical Papilla (SCAP) have aroused great interest in the scientific community due to their relationship with the challenge of treating teeth with incomplete root formation ^1^ ^,^ ^2^. This cell population was initially isolated in 2006 ^3^ and represents a cell type distinct from the pulp, highlighted by its ability to differentiate cells into odontoblasts. Due to their characteristics, these cells have been studied with a particular interest in regenerative Endodontics ^1^ ^,^ ^2^.

The main advantage of developing research using cell culture assays is based on the possibility of investigating the specific role of receptors, cell signaling molecules, enzymes, and mediators by pharmacological blockade ^4^. Such experimental designs are useful for understanding disease mechanisms as well as biomaterials' effects on living cells ^5^ ^,^ ^6^ ^,^ ^7^. These compounds are mainly insoluble, and organic solvents are required for their use at in vitro assays such as Dimethyl sulfoxide (DMSO).

DMSO is a polar organic compound of amphipathic nature. This molecule is widely used as an adjuvant in dissolving insoluble compounds in an aqueous medium, such as those used for a pharmacological blockade ^8^ ^,^ ^9^. A recent study demonstrated that DMSO could induce significant molecular changes in cells originating from cardiac muscle and hepatic tissue. However, the authors also point out possible damages that may occur obeying a tissue-specific profile. ^9^

Today, scientific literature brings relevant information from studies that used human apical papilla cells under distinct methodological contexts. However, the literature still lacks standards for the usage of DMSO in *in vitro* experimental designs. Varying concentrations of DMSO might lead to differential effects on the viability and mineralization of these cells. Considering the relevance of using hAPC cultures for studies focusing on Regenerative Endodontics, the present study aimed to investigate DMSO effects on the viability and mineralization activity in hAPC cultures *in vitro* and to establish standards of maximum concentrations, providing critical insights to avoid biases in future experiments and enhance the reliability of research involving these cells.

## Materials and Methods

### Cell Culture

This study was approved by the local Ethics Committee (Process # 4.376.089). Human APCs, collected from immature permanent third molars from healthy patients (n=3) (aged 16-20 years) and previously characterized for their phenotype and function, were used in this study ^5^. Cells at passage six were cultured in alpha-minimum essential medium (α-MEM) (Sigma-Aldrich, St Louis, MO) with 10% FBS (Gibco Life Technologies, Grand Island, NY) and antibiotics (100 µg/mL penicillin, 100 µg/mL streptomycin, 0.5 mg/mL amphotericin B - Invitrogen) under standard culture conditions (37°C, 100% humidity, 5% CO_2_, and 95% air).

### Cell viability Assay

The viability of hAPCs stimulated with different DMSO concentrations was analyzed through the 3-(4,5-Dimethylthiazol-2-yl)-2,5-diphenyltetrazolium bromide (MTT) assay, following the guidelines outlined in the ISO 10993-12:2012(E). hAPCs were seeded at a density of 1.25 x 10^4^ cells per well in 96-well plates. After an initial 24-hour period, the medium was switched from 10% FBS α-MEM to 1% FBS α-MEM to facilitate cell adaptation. Following another 24-hour incubation, cells were stimulated with different DMSO concentrations (0.1%, 0.5%, 1%, 5%, and 10%; Sigma-Aldrich, St. Louis, MO, USA) in 1% FBS α-MEM, with experiments conducted in septuplicate. Cells kept at 1% FBS α-MEM and 0% DMSO represented the control group.

Cells were cultured for up to 7 days, with the medium replaced every 3 days. At 24 h, 48 h, 72 h, and 7 days time-point, the cell supernatant was replaced with 20 µL of MTT solution (Sigma-Aldrich, St. Louis, MO, USA) (5 mg/mL) in phosphate-buffered saline (PBS) followed by 180 µL of 10% FBS α-MEM. The cells were then incubated at 37°C for 4 h, protected from light. After incubation, the MTT solution was replaced with 100 µL Dimethyl sulfoxide (Synth, Diadema, SP, Brazil). The optical density was measured at a wavelength of 570 nm using a Synergy HT microplate reader (Biotek, Instruments, Inc. Winooski, VT, USA).

### Cell Mineralization Activity Assay

The effect of DMSO (0.1%, 0.5%, and 1%) on the osteo/odontogenic mineralization potential of hAPC was evaluated through the Alizarin red S staining assay. Cells were seeded in triplicate at a density of 1.5 x 10^4^ cells per well in 48-well plates and kept in either a proliferation medium or osteogenic induction medium (proliferation medium supplemented with 2mmol/L KH_2_PO_4_ and 100 nmol dexamethasone) for a period of 21 days. Prior to cell fixation, the Alamar Blue cell viability assay (Cat. DAL1025, Invitrogen) was performed according to the manufacturer's instructions to standardize the data. Subsequently, cells were fixed with 4% paraformaldehyde for 30 minutes, rinsed with PBS, and stained with 40 mmol/L Alizarin red S solution (Cat. A5533, Sigma-Aldrich; pH = 4.2) for an additional 30 minutes. Both macroscopic and microscopic (Nikon Eclipse Ti light microscope; 10x magnification) evaluations were performed for each group. Semi-analytical densitometry analysis of calcium deposits was conducted by solubilizing the stain with 10% ammonium hydroxide solution and measuring absorbance at 405 nm.

### Statistical Analysis

Statistical analyses were conducted using GraphPad Prism 9.0 (GraphPad Software, San Diego, CA, USA). The normality of data was assessed using the Shapiro-Wilk test. Subsequently, a one-way analysis of variance (ANOVA) was performed, followed by Tukey's post hoc test. The significance level was set at p<0.05.

### Results

### Cell Viability

**Figure 1:**
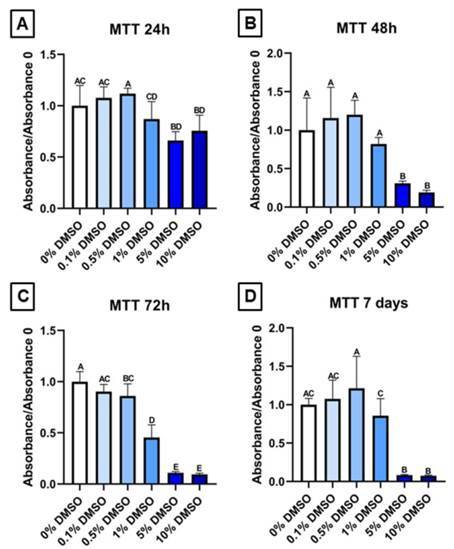
Viability of human Apical Papilla Cells stimulated with Dimethyl sulfoxide. Absorbance/absorbance 0 (0% DMSO - control group) (570nm) data obtained from the MTT assay at experimental times of 24 h (A), 48 h (B), 72 h (C), and 7 days (D) in hAPC exposed to different concentrations of Dimethyl sulfoxide (0%, 0,1%, 0,5%, 1%, 5%, and 10%). The results showed the mean and standard deviation of the experiments performed in septuplicate. Different letters represent statistical differences between groups. (One-Way ANOVA with Tukey's test, p<0.05)

The results of the MTT assay are shown in Figure 1. According to the ISO 10993-5:1999 (E) recommendations, biomaterials and chemical compounds, such as DMSO, that reduce cell viability by more than 30% are considered cytotoxic. In general, 0.1% and 0.5% did not significantly reduce cell viability compared to the control at any time-point (Figure 1A, B, and D), except for 0.5% DMSO at 72 h time-point, which reduced cell viability but not by more than 30%, and was thus not considered cytotoxic (Figure 1C). The 1% DMSO concentration did not show cytotoxicity at earlier time-points, but significantly reduced cell viability at the 72 h and 7 days, indicating cytotoxicity at 72 hours (Figure 1C and D). The 5% DMSO concentration was cytotoxic to hAPC at all analyzed experimental time points, consistently reducing cell viability by more than 30% (Figure 1A, B, C, and D). The 10% DMSO concentrations reduced cell viability at all time points, showing cytotoxicity from 48 hours onwards (Figure 1A, B, C, and D).

### Cell Mineralization Activity

**Figure 2:**
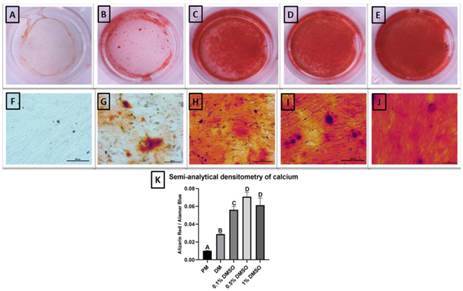
Human Apical Papilla Cells mineralization activity. Alizarin red S staining assay was performed to analyze the calcium deposition of hAPC exposed to different concentrations of Dimethyl sulfoxide (0.1%, 0.5%, and 1%) in the 21-day time-point. Cells under proliferation medium (A and F). Cells under differentiation medium (B and G). Cells under DMSO 0.1% (C and H), 0.5% (D and I), and 1% (E and J). Macroscopic view (A-E). Microscopic view (F-J). Semi-analytical densitometry of calcium standardized by the Alamar Blue (J). The results showed the mean and standard deviation of the experiments performed in triplicate. Different letters represent statistical differences between groups (One-Way ANOVA with Tukey's test, p<0.05).

Human APC DMSO-stimulated presented higher osteo/odontogenic mineralization potential compared to the control group (DM) (Figure 2). Qualitative results showed an increase in mineralization in a DMSO concentration-dependent manner. The higher DMSO tested concentration (1%) showed higher calcium nodules production under both microscopic (Figure 2J) and macroscopic view (Figure 2E). The semi-analytical densitometry of calcium pointed out that 0.1% DMSO is significantly higher than the control group and 0.5% and 1% DMSO do not present differences between them but showed significantly higher than the control group and the 0.1% DMSO group (Figure 2K).

## Discussion

Cell culture has been an essential tool for scientific research. Two-dimensional (2D) cell culture is an in vitro model that is frequently used to develop studies that aim to understand cell behaviors in vivo or cellular responses to external factors such as drugs. Drug-response assays are widely performed in 2D cellular cultures to evaluate drug efficacy, potency, or toxicity in specific cell types ^10^. The main advantage of using in vitro experimental models lies in the possibility of investigating the role of specific molecules such as proteins, growth factors, receptors, enzymes, and cell signaling proteins through their blockade. For this purpose, experimental tools such as small-interfering RNAs, neutralizing antibodies, and selective inhibitors might be considered. Pharmacological inhibitors might be conveniently used since they are properly prepared according to manufacturers' instructions ^5^ ^,^ ^6^ ^,^ ^11^.

DMSO is an aprotic solvent used to solubilize nonpolar or poorly soluble polar molecules and drugs due to its amphipathic nature. ^12^ Drugs extensively studied in vitro as indomethacin ^13^ ^,^ ^14^, losartan ^15^, anti-cancer drug cisplatin ^16^, and a representative number of antibacterial drugs, including tetracyclines, polymyxins, fluoroquinolones as well as β-lactams ^17^ have DMSO as a solvent.

In addition, its application in the cryopreservation of cells is also consolidated, but it depends on high concentrations, 7% to 10% ^9^. Literature shows that these concentrations prevent cell death during the freezing process and do not interfere with hAPC potential regarding cell viability, multilineage differentiation, colony-forming efficiency, cell proliferation, or profile mesenchymal stem cell markers. ^18^

However, when it comes to DMSO used directly on the cells, the literature shows that it can cause damage in different cell lineages. DMSO treatment in cochlear culture activated caspase-9, caspase-8, and caspase-3, suggesting that signaling cell death through membrane mitochondria starts apoptosis. In addition, DMSO also induced cell death through caspase-9 activation in the EL-4 cell line ^19^. DMSO concentrations up to 1% also revealed cytotoxics to the retinal neuronal cell line; concentrations up to 10% were cytotoxics through plasma membrane pores formation, while low concentrations of 2% to 4% involved apoptosis-inducing factor translocation from mitochondria to the nucleus and poly-(ADP-ribose)-polymerase activation ^12^. Another study showed that DMSO 0.1% induced significant molecular changes involving cardiac and hepatic tissues through transcriptome, proteome, and DNA methylation profiles ^9^.

The DMSO effects on hAPC culture were still not known, and our study aimed to evaluate the maximum DMSO doses allowed to these lineages in terms of cell viability. The results showed through MTT assay that 5% and 10% DMSO concentrations were shown cytotoxic for hAPC at all the analyzed experimental time-points, revealing that these concentrations should be avoided for drug dilutions. The concentrations of 0.1% and 0.5% DMSO might be considered safe regarding cell viability for experimental periods until 7 days at 1% FBS. Despite that, once performing studies using pharmacological inhibitors eluted at DMSO, it seems reasonable to highlight the relevance of including the DMSO-containing control group with the corresponding concentration. This precaution is mandatory to avoid eventual experimental biases due to the toxicity of DMSO traces.

The MTT assay is widely regarded for cell viability determination and is a reliable indicator of metabolic activity ^20^. While it presents some limitations, such as not differentiating between cell cycle or metabolism alterations, anti-proliferative effects, apoptosis, or necrosis, the MTT assay was well-suited for our study to assess the overall viability and metabolic activity of hAPC. A significant difference in the MTT results already provides a clear indication of the effects of DMSO on hAPC. Although other cell viability assays, such as the Crystal Violet assay, are typically used to complement MTT results, their limitations made them inappropriate for our experimental design. The Crystal Violet assay, for example, can result in non-specific staining of dead cells, leading to inaccurate measurements of viable cell numbers, especially when cultures reach confluence ^21^. Given our study's long culture times and resulting confluent cells, the Crystal Violet assay would have been unsuitable.

Our results also showed that DMSO increased osteo/odontogenic mineralization in hAPC. Although it is known cell death leads to calcium deposits ^22^, the standardization of our data by the Alamar Blue cell viability assay allows us to confirm that the calcium deposits are not a consequence of higher cell death in DMSO groups. The findings underscore the importance of incorporating a control group containing DMSO at the same concentration in studies assessing hAPC mineralization activity to prevent potential experimental biases arising from DMSO's capacity to enhance mineralization in hAPC cultures. The methodology that was employed to determine mineralization, Alizarin Red staining, cannot differentiate between physiological and dystrophic calcification ^23^. Mechanisms related to these types of calcification should be acknowledged in future studies, as this vehicle might impact the mineralization activity of the cells.

## Conclusion

In conclusion, DMSO is clearly cytotoxic at concentrations of 5% and higher. Ideally, it should not exceed 0.5% of the total medium volume. Additionally, DMSO has the potential to enhance mineralization in hAPC cultures, and, despite that, mineralization activity assay should be designed in order to avoid biases.
